# Comparing the Biology of Young versus Old Age Estrogen-Receptor-Positive Breast Cancer through Gene and Protein Expression Analyses

**DOI:** 10.3390/biomedicines11010200

**Published:** 2023-01-12

**Authors:** Alaa Siddig, Wan Faiziah Wan Abdul Rahman, Siti Norasikin Mohd Nafi, Sarina Sulong, Maya Mazuwin Yahya, Tengku Ahmad Damitri Al-Astani Tengku Din, Rozaimi Razali, Kamarul Imran Musa

**Affiliations:** 1 Department of Pathology, School of Medical Sciences, Universiti Sains Malaysia, Kelantan 16150, Malaysia; 2 Breast Cancer Awareness & Research Unit, Hospital Universiti Sains Malaysia, Kelantan 16150, Malaysia; 3 Human Genome Centre, School of Medical Sciences, Universiti Sains Malaysia, Kelantan 16150, Malaysia; 4 Department of Surgery, School of Medical Sciences, Universiti Sains Malaysia, Kelantan 16150, Malaysia; 5 Department of Chemical Pathology, School of Medical Sciences, Universiti Sains Malaysia, Kelantan 16150, Malaysia; 6 Department of Biomedical Sciences, College of Health Sciences, QU-Health, Qatar University, Doha P.O. Box 2703, Qatar; 7 Department of Community Medicine, School of Medical Sciences, Universiti Sains Malaysia, Kelantan 16150, Malaysia

**Keywords:** breast cancer, young age, gene expression, estrogen-receptor-positive, transcriptomic profile, *GLYATL-1*, *RANBP3L*

## Abstract

Background: Breast cancer developed at a young age (≤45 years) is hypothesized to have unique biology; however, findings in this field are controversial. Methods: We compared the whole transcriptomic profile of young vs. old-age breast cancer using DNA microarray. RNA was extracted from 13 fresh estrogen receptor (ER)-positive primary breast cancer tissues of untreated patients (7 = young age ≤45 years and 6 = old age ≥55 years). In silico validation for the differentially expressed genes (DEGs) by young-age patients was conducted using The Cancer Genome Atlas (TCGA) database. Next, we analyzed the protein expression encoded by two of the significantly down-regulated genes by young-age patients, Glycine N-acyltransferase-like 1 (*GLYATL-1*) and Ran-binding protein 3 like (*RANBP3L*), using immunohistochemical analysis in an independent cohort of 56 and 74 ER-positive pre-therapeutic primary breast cancer tissues, respectively. Results: 12 genes were significantly differentially expressed by young-age breast cancers (fold change >2 or <2- with FDR *p*-value < 0.05). TCGA data confirmed the differential expression of six genes. Protein expression analysis of GLYATL-1 and RANBP3L did not show heterogeneous expression between young and old-age breast cancer tissues. Loss of expression of GLYATL-1 was significantly (*p*-value 0.005) associated with positive lymph node status. Higher expression of RANBP3L was significantly associated with breast cancers with lower histopathological grades (*p*-value 0.038). Conclusions: At the transcriptomic level, breast cancer developed in young and old age patients seems homogenous. The variation in the transcriptomic profiles can be attributed to the other clinicopathological characteristics rather than the age of the patient.

## 1. Introduction

Breast cancer developed at a young age (≤45 years) has aggressive clinicopathological characteristics and poor clinical outcomes compared to breast cancer developed at old age [1,2,3,4,5,6,7,8]. An earlier gene expression study revealed that breast cancer at a young age (≤45 years) differentially expresses more than 350 gene sets compared to breast cancer in older women (≥65 years) [9]. The research group reanalyzed the exact data and found that breast cancer at a young age has a higher proportion of aggressive intrinsic subtypes rather than a distinct transcriptomic profile, and thereby recommended that treatment options must rely on the tumor grade and molecular subtype and not on the age of the patient [10]. In contrast, a literature-based study reported that 12 genes were expressed in an age-related manner in breast cancer tissue; the upregulated genes were involved in biological processes associated with immature mammary cell populations and growth factor signaling, while the downregulated genes were related to apoptotic pathways, suggesting that young patients may benefit from targeted therapy [11]. Johnson et al. also found that several genes for proliferation, invasion, and metastasis show differential expression between young (<40 years) and old (>40 years) age breast tumors. In addition, the expression of those genes appears to correlate with patient prognosis [12]. Analyzing a large cohort of breast cancer gene expression data, Liao et al. found that the distinct genomic profile of breast cancer at a young age (≤45 years) can only be observed in estrogen-receptor (ER)-positive cancer, whereas in ER-negative tumors, the unique biology vanishes [13]. Using DNA microarray analysis, Malvia and colleagues found that numerous genes implicated in breast cancer invasion and metastasis were significantly upregulated in early-onset breast cancer (≤40 years) compared to late-onset breast cancer (≥55 years) [14]. A recent study analyzed publicly available gene expression data sets of primary breast cancer. The study reported that young-age breast cancers are enriched with cell proliferation gene signatures that have strong prognostic values; these findings were observed mainly in luminal subtypes [15].

With all these controversial findings regarding the biology of breast cancer at a young age, further research in light of previous literature is warranted. The current study focused on ER-positive cancer as those tumors encompass more than 70% of all breast cancers [16]. In addition, previous findings emphasized that the gene expression profile of ER-negative tumors does not show variation among different age groups [13]. Furthermore, epidemiological studies highlighted that among ER-positive breast cancer patients, age remains an independent prognostic factor for clinical outcomes [7,8].

## 2. Materials and Methods

### 2.1. Breast Cancer Tissues

We collected 13 fresh ER-positive primary breast cancer tissues from untreated patients (7 = young age ≤45 years and 6 = old age ≥55 years). Breast tissue samples were transferred immediately from the operation theatre to Pathology Laboratory, Hospital Universiti Sains Malaysia (HUSM) for further analysis. Less than approximately 50 mg of tissue was collected from the tumor area by a well-trained pathologist. Tumor tissues were placed immediately in Paxgene Fixative solution (PreAnalytiX, Hombrechtikon, Zürich, Switzerland) and kept overnight at 4 °C. After 24 h, the tissues were transferred to Paxgene stabilizer solution (PreAnalytiX, Hombrechtikon, Zürich, Switzerland) and kept at −80 °C until the RNA extraction procedure. Paxgene fixative can preserve tissue morphology and nucleic acid integrity. Breast cancer tissues were used in agreement with the ethical approval obtained from the Human Research Ethics Committee (HREC), Universiti Sains Malaysia, which complies with the declaration of Helsinki, Study protocol code USM/JEPeM/19090547, approved on the 2 January 2020.

### 2.2. RNA Extraction 

Less than 50 mg of breast cancer tissue was used for RNA extraction. Total RNA was extracted using TRIzol reagent (Sigma-Aldrich, St Louis, MO, USA) and RNeasy Mini Kit (QIAGEN, Hilden, Germany), following manufacturer instructions with minimal modifications. Quality was assessed using Agilent 2100 Bioanalyzer (Thermo Fisher Scientific, Waltham, MA, USA). RNA samples with RNA integrity number (RIN) ≥7 indicated good quality RNA. In our study, 9 out of 13 samples had RIN above 7, and 4 samples had RIN numbers below 7 (sample no. 5 = 6.8, sample no. 17 = 6.4, and sample no. 19 = 6.80), which were also used for transcriptomic profiling. One sample from young age patients was excluded from further analysis as the RIN number was 3.90. 

### 2.3. Transcriptomic Profiling

Gene expression profiling was performed using the Affymetrix Clariom S Human microarray gene chip. The gene chip contains probe sets that interrogate more than 21,000 well-annotated genes. In summary, 125 ng RNA was processed by a Whole Transcript (WT) Plus reagent kit (#902280) from Thermo Fisher Scientific to synthesize the cDNA. Following, cDNA was fragmented and labeled. Labeled cDNA was added to the hybridization mix and incubated in a Clariom S human array for 16 h at 45 °C. Subsequently, the Gene Chip FS450 fluidics station was used to stain and wash the cartridges. Finally, the Gene Chip Scanner 3000 7G was used to scan the cartridges. Raw data were imported to transcriptome analysis console (TAC) software (version 4.0.2.15) for further analyses. 

### 2.4. Differential Gene Expression Analysis 

Gene expression analysis was performed using TAC software. The CEL files were normalized and summarized using the robust multi-array analysis (RMA) algorithm [17]. The Limma package was used to identify the differentially expressed genes (DEGs) between young and old-age breast cancers. 

### 2.5. In Silico Validation for the Differentially Expressed Genes by Young Age Breast Cancer Tissues

The expression of the 12 differentially expressed genes by young-age breast cancer was validated using the web-portal UALCAN http://ualcan.path.uab.edu (accessed on 12 December 2022) [18], a database that utilizes The Cancer Genome Atlas (TCGA) gene expression data. 

### 2.6. Analysis of Prognostic Significance of the Differentially Expressed Genes by ER-Positive Young-Age Breast Cancer

A Kaplan-Meier plotter for breast cancer (http://kmplot.com/analysis/, accessed on 12 December 2022) was used to assess the prognostic value of the DEGs by young-age patients. The relapse-free survival curves were presented with hazard ratio and 95% confidence intervals [19].

### 2.7. Protein Expression Analysis Using Immunohistochemical (IHC) Staining 

IHC staining was performed to measure the change in protein expression associated with the change in mRNA levels. Two significantly differentially expressed genes by young-age breast cancer tissues were selected (*GLYATL-1* and *RANBP3L*) (Fold change = −21.37, −14.28, False Discovery Rate (FDR) *p*-value 0.033, 0.0247), respectively. Both genes showed loss of expression in young age compared to old age cancers in the current gene expression study. Antibodies against the encoded proteins were applied to the formalin-fixed paraffin-embedded tissue sections of an independent larger breast cancer cohort. 

#### 2.7.1. Breast Cancer Tissue Blocks

IHC analysis was carried out at the Pathology laboratory, Universiti Sains Malaysia, Kelantan, Malaysia. Surgical and core biopsy tissue blocks of breast carcinoma patients who were diagnosed histopathologically in the period between January 2013 and June 2022 were retrieved. The inclusion and exclusion criteria for the protein expression study matched those mentioned earlier for gene expression analysis (positive ER status, age at diagnosis ≤45 years or ≤55 years, pre-therapeutic samples). In addition, patients with unavailable tissue blocks or who had tissue blocks but with insufficient material for immunohistochemical analysis were excluded. A total of 74 patients were included. However, later on, 74 samples were successfully stained with RANBP3L and 57 samples were stained for GLYATL-1 due to limited resources. The corresponding demographic and clinicopathological features were recorded, including the patient’s age, tumor size, histopathological grade, histopathological subtype, lymph node positivity, progesterone receptor (PR) status, and HER-2 amplification status by IHC and equivocal cases were confirmed by the dual– scolor hapten brightfield in situ hybridization method (DDISH). 

#### 2.7.2. Immunohistochemical Staining 

3 µm formalin-fixed paraffin-embedded (FFPE) tissue sections were cut on poly L lysine slides using a microtome (LEICA RM 2245). The sections were dewaxed and hydrated using xylene and gradient ethanol. Then, slides were immersed in a target antigen retrieval solution (pH 6.0, citrate buffer) in a pressure cooker for 30 min to retrieve the masked epitopes. Next, sections were incubated with 3% hydrogen peroxide for 10 min to block the endogenous peroxidase activity. Then, for each patient, one section was incubated with Rabbit polyclonal GLYATL-1 primary antibody (Abcam, UK, cat No: ab187859, 1:100 dilution) and one was incubated with Rabbit polyclonal RANBP3L primary antibody (Abbexa, cat No: abx027590, 1:100 dilution); the incubation period for both antibodies was overnight at 4 °C. The next day, slides were washed twice with Tris Buffered Saline (TBS) and incubated with horse reddish peroxidase (Dako REAL EnVision™+System, cat No: K5007) for 30 min at room temperature. To visualize the antigen-antibody reaction, slides were incubated with 3, 3′-diaminobenzidine tetrahydrochloride chromogen for 5 min. Finally, slides were washed with the TBS and stained with freshly prepared hematoxylin for 5 min. Finally, sections were dehydrated, cleared, and mounted. 

As recommended by the manufacturer, kidney tissue sections were used for positive and negative controls for both antibodies. In case of negative control the primary antibody incubation step was omitted.

#### 2.7.3. IHC Scoring

The H-score system was used for both antibodies. This scoring system depends on determining the intensity of the stain (0, no staining; 1, weak staining; 2, mild to moderate staining; 3, strong staining) and the percentage of positive tumor cells. The following equation was used to calculate the score [20].

H-score = (% of cells with weak staining × 1) + (% of cells with moderate staining × 2) + (% of cells with strong staining × 3). 

The resulting H-score ranged from 0 to 300. An H-score of more than the median value (200 for both markers) was established as a cut-off value to distinguish high expression from low expression. The positive control section of the GLYATL-1 antibody needed to show cytoplasmic immunoreactivity, whereas nuclear and cytoplasmic immunoreactivity was required for the RANBP3L antibody. All slides were scored by two independent observers who were blinded to the study design and the clinicopathological features of included cases.

### 2.8. Statistical Analysis

A one-way ANOVA test was used to determine the differentially expressed genes between young and old-age breast cancers. The estimated variance by the ANOVA test for each probeset was corrected using the information from other probesets (eBayes empirical parameter) [21]. Using eBayes is mandatory in studies utilizing a small sample size. The moderated t-statistics test was used to calculate the *p*-value for each transcript cluster (gene). To control the false discovery rate resulting from multiple testing, Benjamini- Hochberg’s method was applied. Each gene with a fold change >2 or <−2 with an adjusted *p*-value of less than 0.05 was considered significantly differentially expressed.

For the protein expression, data analysis was performed using SPSS software version 26.0. Descriptive statistics were used to describe the clinicopathological characteristics of the included cases. Variables were presented as frequency (*n*) and percentage (%). Associations between GLYATL-1 and RANBP3L expression and various clinicopathological characteristics were assessed using Pearson’s chi-square test and Fisher’s exact test. An association with a *p*-value less than 0.05 was considered significant.

## 3. Results

### 3.1. Patients and Clinicopathological Characteristics of Breast Cancer Samples

From July 2020 to May 2021, 19 breast cancer tissue samples from untreated patients were collected at the histopathology laboratory at Hospital Universiti Sains Malaysia (HUSM). Of these 19 samples, 13 patients met the inclusion criteria. Of the 13 cases, 7 were ≤45 years of age and 6 were ≥55 years. All patients were diagnosed histopathologically with invasive breast carcinoma of no specific type except one mucinous-type case. All the tumors were ER-receptor positive by IHC analysis. Later on, one patient from the young age group was excluded due to the poor quality of the extracted RNA (RNA Integrity Number 3.9). Accordingly, a total of 12 patients were successfully enrolled in the gene expression study. The clinicopathological characteristics of the patients included in the gene expression study are summarized in Table 1.

### 3.2. Differentially Expressed Genes

**Figure 1:**
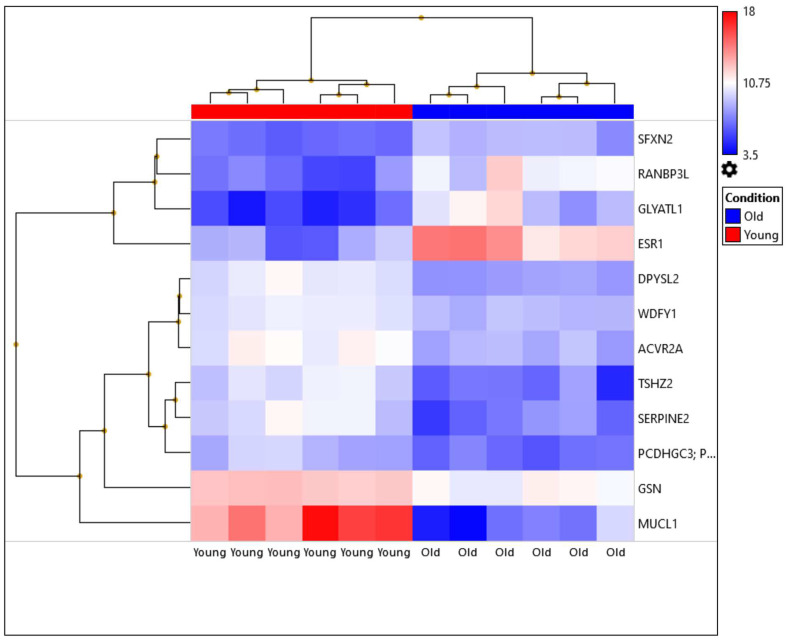
Heatmap illustrating the expression levels of the differentially expressed genes (DEGs) between the young and old-age breast cancer tissues (*n* = 12). Hierarchical clustering of the 12 ER-positive breast cancer cases. Six samples of young age patients ≤45 years and six samples of old age patients ≥55 years. The expression levels for each differentially expressed gene are reflected by a color ranging from blue (low) to red (high).

Out of 21,448 tested genes, only 12 genes were significantly differentially expressed in young-age breast cancer (fold change of <2 or >2 and FDR *p*-value < 0.05). Of these differentially expressed genes, 8 were upregulated (*DPYSL2*, *MUCL1*, *GSN*, *ACVR2A*, *TSHZ2*, *SERPINE2*, *WDFY1*, and *PCDHGB7*), while 4 were down-regulated (*SFXN2*, *RANBP3L*, *ESR1*, and *GLYATL1*). The level of expression of the DEGs (*n* = 12) is illustrated in Table 2 and Figure 1. A summary regarding the significance of the DEGs in tumorigenesis and cancer progression is presented in Table 3.

### 3.3. In Silico Validation of the Differentially Expressed Genes by Young-Age Breast Cancer 

**Figure 2:**
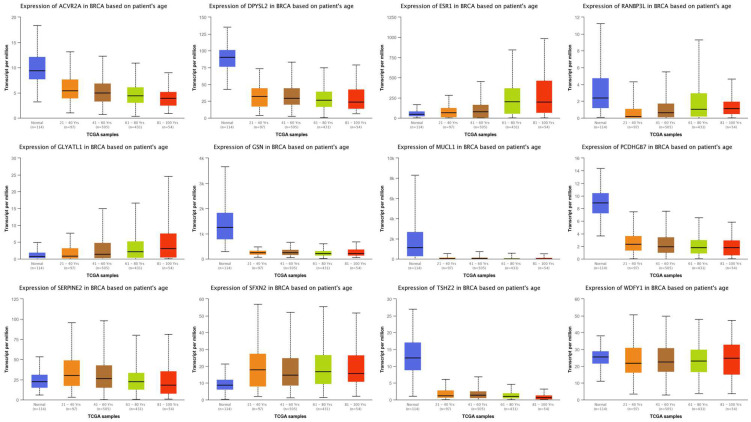
Validation of the differentially expressed genes by young-age breast cancer tissues using RNA-seq data from TCGA database. *ACVR2A*, *SERPINE2*, and *PCDHGB7* show upregulation in young-age breast cancer patients compared to old age patients. *RANBP3L*, *GLYATL-1*, and *ESR1* show down-regulation in young age patients compared to old age patients. *p*-values for each comparison are presented in Supplementary Table S1.

To validate the expression levels of the DEGs in a larger sample size, we used the web portal UALCAN http://ualcan.path.uab.edu [18], a database that utilizes The Cancer Genome Atlas (TCGA) gene expression data. Breast cancer expression data were used and a filter was set to determine the expression of the 12 genes among different age groups in breast cancer. Six genes out of twelve showed consistent expression (*RANBP3L*, *GLYATL1*, ESR1, *ACVR2A*, *SERPINE2*, and *PCDHGB7*) (Figure 2).

### 3.4. Prognostic Significance of the Differentially Expressed Genes by Young Age ER-Positive Breast Cancer

**Figure 3:**
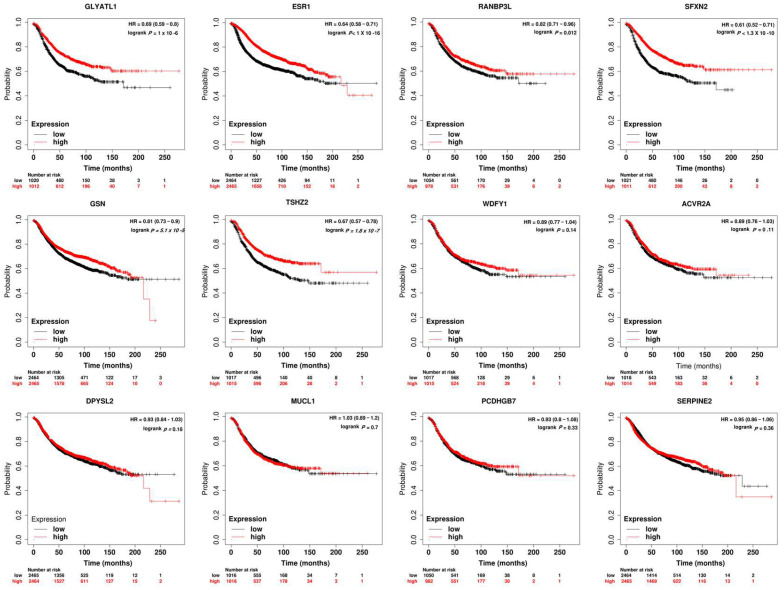
Prognostic Significance of the differentially expressed genes by ER-positive young-age breast cancer. Lower expression of *RANBP3L*, *GLYATL1*, *ESR1*, and *SFXN2* were significantly related to shorter relapse-free survival (RFS). However, high expressions of *GSN* and *TSHZ2* were significantly related to longer RFS.

Survival analysis showed that the loss of expression of *RANBP3L*, *GLYATL1*, *ESR1*, *and SFXN2* was significantly related to shorter relapse-free survival (RFS). High expressions of *GSN* and *TSHZ2* were significantly related to longer RFS (Figure 3).

### 3.5. Evaluation of GLYALT-1 and RANBP3L Protein Expression Using Immunohistochemical Analysis 

The clinicopathological characteristics of breast cancer patients included in the immunohistochemical analysis of GLYALT-1 and RANBP3L (*n* = 56 and 73 patients, respectively) included age, tumor size, histopathological grade, histopathological subtype, lymph node positivity, progesterone receptor status and HER-2 expression status. These characteristics are summarized in Table 4 and Table 5.

#### 3.5.1. GLYATL-1 Expression in Breast Carcinoma Tissues 

**Figure 4:**
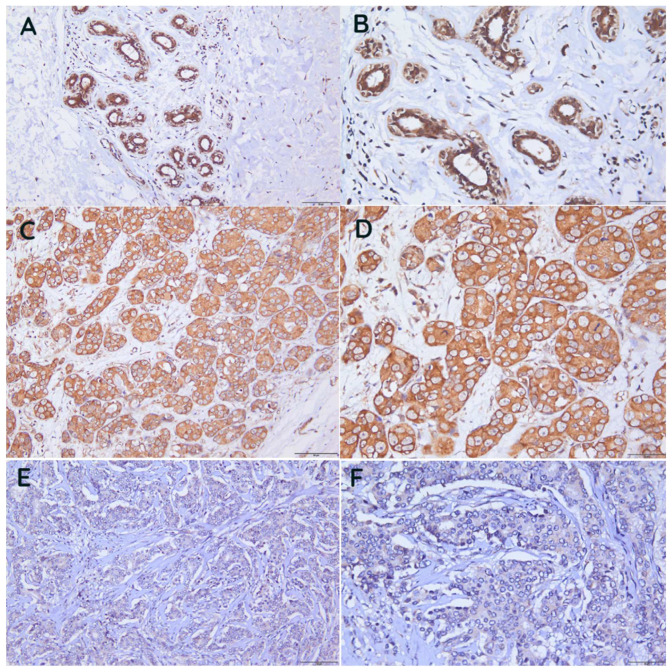
GLYATL-1 expression in normal breast duct, breast carcinoma in situ, and invasive breast carcinoma tissues. GLYATL-1 expression in (**A**,**B**) normal breast duct tissues. (**C**,**D**) Breast carcinoma in situ. (**E**,**F**) Invasive breast carcinoma tissues. (**A**,**C**,**E**) ×20 μm. (**B**,**D**,**F**) ×40 μm.

An immunohistochemical stain of GLYATL-1 was performed for 56 breast carcinoma tissue sections. The expression of GLYATL-1 was localized to the cytoplasm. Higher expression of GLYATL-1 was observed in 41.1% of the sample (23/56), whereas 58% (33/56) of cases exhibited a low level of expression. Normal breast ducts showed a higher expression of GLYATL-1 compared to breast carcinoma in situ and invasive breast carcinoma tissues (Figure 4).

#### 3.5.2. Association between GLYATL-1 Expression with the Age and Other Clinicopathological Parameters of Patients with Breast Carcinoma

Lower expression of GLYATL-1 was strongly associated with positive lymph node status (*p* < 0.005 obtained by Fisher’s exact test). However, no significant association was found between GLYATL-1 expression and the other clinicopathological features including: age, tumor size, histopathological grade, histopathological subtype, PR and HER-2 status (see Table 6).

#### 3.5.3. Expression of RANBP3L in Breast Cancer Tissues 

**Figure 5:**
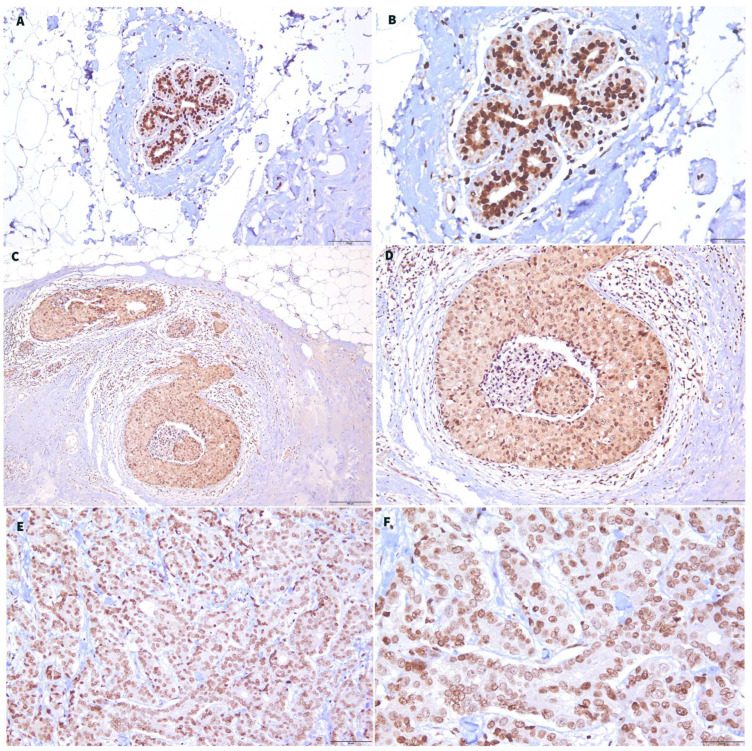
RANBP3L expression in normal breast duct, ductal carcinoma in situ, and invasive breast carcinoma tissues. RANBP3L expression in (**A**,**B**) normal breast duct tissues, (**C**,**D**) ductal carcinoma in situ tissues, and (**E**,**F**) invasive breast carcinoma tissues; (**A**,**E**,**F**), ×50 μm, (**B**), ×20 μm, (**C**,**D**), ×100 μm.

The expression level of RANBP3L was examined in 74 breast carcinoma tissue sections. The signal we obtained was nuclear and cytoplasmic (Figure 5). A high level of RANBP3L expression was observed in 64% of the cases (47/74), whereas 36% of cases (27/74) exhibited a low level of expression. A strong nuclear signal was observed in normal breast ducts, whereas moderate nuclear and cytoplasmic signals were observed in ductal carcinoma in situ cases. The signal faded with a higher histopathological grade (Figure 5).

#### 3.5.4. Association between RANBP3L Expression and Age and Other Clinicopathological Parameters of Patients with Breast Carcinoma

Higher expression of RANBP3L was significantly associated with a lower histopathological grade (Grade 1) (*p* < 0.038 obtained by Fisher’s exact test); however, no significant association was found between RANBP3L expression and the other clinicopathological features such as: age, tumor size, histopathological subtype, PR and HER-2 status (see Table 7).

## 4. Discussion

Management of breast cancer developed at a young age is considered a clinical dilemma as breast cancer at a young age has poor clinicopathological characteristics at the time of presentation compared to breast cancer that arises in old age [36,37]; in addition, locoregional recurrence is a more frequent event in young age patients [38]. Furthermore, young age has been proposed as an independent factor for shorter breast cancer-specific and overall survival [39,40]. 

In the current study, we compared the transcriptomic profile of young (≤45 years) versus old age (≥55 years) ER-positive breast cancers of untreated patients. The differential gene expression analysis revealed that 12 genes were differentially expressed by young-age breast cancer tissues (fold change >2 or <−2 with FDR *p*-value < 0.05). Of the 12 genes, 8 were upregulated and 4 were downregulated.

*RANBP3L* and *GLYATL-1* showed extreme loss of expression in young age patients; this finding is consistent with Liao and colleagues’ findings. They found that ER-positive breast cancer in pre-menopausal women (≤45 years) has a lower level of *RANBP3L* and *GLYATL-1* compared to post-menopausal women (≥55 years). It is noteworthy that Liao et al.’s study included more than 2500 breast cancer cases [13]. The protein encoded by *RANBP3L* is a nuclear exporter of bone morphogenetic protein-specific smads and plays a critical role in mesenchymal stem cell differentiation [41]. Loss of *RANBP3L* expression was found to be associated with loss of epithelial differentiation and induced cell migration behavior in renal cancer cells [26]. However, loss of *GLYATL-1* expression was significantly associated with higher Gleason scores in prostatic adenocarcinoma [31], with shorter overall survival in hepatocellular carcinoma patients [30].

Estrogen receptor 1 *ESR1* also showed down-regulation in young age patients compared to old age patients; this is in line with a previous large-scale genomic analysis that revealed that breast cancer developed in young age patients has a lower level of *ESR1* mRNA compared to breast cancer in old age patients [9]. Loss of *ESR1* expression is characteristic of acquiring resistance to chemotherapy in ER-positive breast cancer [42]. In a previous study, poor clinical outcomes were observed in young age patients who both received and did not receive tamoxifen therapy. This is a clear indication of endocrine therapy resistance in this group of patients [7]. 

Another noteworthy significantly down-regulated gene by young age patients in the present study was sideroflexin 2 (*SFXN2)*; the encoded protein by *SFXN2* is implicated in mitochondrial iron metabolism [43]. Loss of this protein may result in abnormal iron metabolism which, in turn, may result in tumor initiation, progression, and metastasis [24].

Survival analyses in the present study revealed that loss of *RANBP3L*, *GLYATL-1, ESR1,* and *SFXN2* was significantly related to lower RFS in breast cancer (*p*-value < 0.012, 1.1 × 10^−6^, 1 × 10^−16^, and 1.3 × 10^−10^, respectively).

One of the genes that showed significant overexpression by the young age group was mucin-like 1 (*MUCL1)* (FC = 448.69, FDR-*p*-value < 0.009). Previous studies found that *MUCL1* is expressed by the majority of breast cancer cell lines (>90%) as it is recognized that mucins in general form the ductal surfaces of several organs, including the breast [44]. Overexpression of *MUCL1* mRNA was strongly correlated with higher tumor grade, lymph node positivity, and high recurrence and death rates in breast cancer patients [23].

We also validated the expression of the 12 DEGs between young and old-age breast cancer tissues using the TCGA database. The results show that six genes have concordance expression to that obtained in our gene expression experiment. The TCGA data showed that young-age breast cancer patients have a lower level of expression of *RANBP3L*, *GLYATL-1*, and *ESR1*, whereas *ACVR2A*, *SERPINE2*, and *PCDHGB7* have higher expression in young-age breast cancer patients compared to old age patients.

There is growing evidence showing that genes that show significant differential expression tend to correlate with the expression of their encoded protein [45,46]. Thus, we selected *RANBP3L* and *GLYATL-1* to compare the expression of their encoded proteins in breast carcinoma tissue samples and determine the relationship between the expression of the proteins and the age of breast cancer patients. The expression of both markers has not been comprehensively investigated previously regarding breast cancer. For *GLYATL-1*, only one published report described the protein expression in clinical breast cancer samples [47]. For *RANBP3L*, to our knowledge, this is the first study to examine the mRNA and protein expression of *RANBP3L* in breast cancer clinical samples.

Protein expression analysis results revealed a strong association between the loss of GLYATL-1 expression and breast tumors with positive lymph node status (*p*-value 0.005). On the other hand, a significant association was found between the high expression of RANBP3L and breast tumors with a low histopathological grade (Modified Bloom Richardson Grade I). However, no significant association was found between the expression of both proteins and the age of breast cancer patients. 

These findings indicate that the difference in the transcriptomic profiles we determined in the current study originate from the poor clinicopathological characteristics of breast cancer at a young age, such as positive lymph node status and a higher histopathological tumor grade, rather than the age of the patient. To verify this, we reanalyzed the gene expression data after matching the lymph node status/histopathological grade. The differentially expressed gene list (*n* = 12) diminished to zero, indicating that the genes that showed differential expression between the two groups were associated with the lymph node positivity status and tumor histopathological grade rather than the age of the patients.

Our findings strongly support an earlier report published by the American Journal of Clinical Oncology verifying that age alone does not add a layer of complexity to the transcriptomic profile of breast cancer but that molecular subtype and tumor grade are the main factors that alter the transcriptomic profile of breast cancer. This study emphasized that treatment options must be selected based on tumor molecular subtype and grade and not patient age [10]. Moreover, an earlier gene expression study involving 99 breast cancer tissue samples concluded that breast cancer gene expression patterns show a strong association with ER status; (this was standardized in the current study), and moderate association with tumor grade; however, no association was found with patient age (<50 years vs. ≥50 years) [48].

Our study limitation is the small sample size used in the gene expression study as a result of using strict inclusion and exclusion criteria; only patients with ER-positive tumors were included as ER-negative tumors do not show differences in the transcriptomic profiles or clinical outcomes among different age groups [8,13]. Additionally, patients who received neoadjuvant therapy were also excluded since it is well-established that neoadjuvant therapy alters the transcriptomic profile of breast cancer [49]. Finally, patients aged between 45–55 years were excluded as they were considered of intermediate age, not belonging to young or old age groups.

Although our study findings do not support the historical hypothesis that breast cancer developed at a young age has unique biology, our findings alter the direction of future research toward other factors that may play a significant role in the poor clinical outcomes of early-onset breast cancer.

## 5. Conclusions

Our findings indicate that the difference in the transcriptomic profiles between young and old age estrogen-receptor-positive breast cancer is due to the aggressive clinicopathological feature of breast cancer developed in young age patients rather than the age of the patients.

## Figures and Tables

**Table 1:** Clinicopathological characteristics of breast cancer cases included in the gene expression study.

Patients ID	Age/Year	Age Group	Tumor Size/cm	TNM Stage	Bloom Richardson Grading	Lymph Node Status	Histological Subtype	ER	PR	HER-2
2	58	Old	2 to 5	pT1N1	Grade 2	Positive	IDC-NST	Positive	Positive	3+
3	82	Old	2 to 5	Unknown	Grade 1	Negative	Mucinous	Positive	Positive	1+
5	45	Young	2 to 5	pT2N3a	Grade 2	Positive	IDC-NST	Positive	Positive	3+
7	39	Young	>5 cm	pT3N0Mx	Grade 3	Negative	IDC-NST	Positive	Negative	3+
8	43	Young	2 to 5	PT2N2a	Grade 2	Positive	IDC-NST	Positive	Positive	3+
9	67	Old	<2 cm	pT1cN3a	Grade 2	Positive	IDC-NST	Positive	Positive	1+
10	45	Young	2 to 5	pT2N1	Grade 3	Positive	IDC-NST	Positive	Positive	3+
12	60	Old	>5 cm	Unknown	Grade 1	Negative	IDC-NST	Positive	Positive	1+
13	38	Young	<2 cm	pT1N2aMx	Grade 2	Positive	IDC-NST	Positive	Positive	3+
17	69	Old	<2 cm	pT2pN0	Grade 3	Negative	IDC-NST	Positive	Negative	3+
18	45	Young	<2 cm	pT2N2aMx	Grade 2	Positive	IDC-NST	Positive	Positive	1+
19	61	Old	>5 cm	pT3N3a	Grade 3	Positive	IDC-NST	Positive	Positive	1+

The expression of estrogen receptor (ER), progesterone receptor (PR), and HER-2 was assessed by immunohistochemical staining. Equivocal HER-2 expression was confirmed using the dual-color dual-hapten brightfield in situ hybridization method (DDISH), Invasive ductal carcinoma- no specific type (IDC-NST).

**Table 2:** List of the significantly differentially expressed genes by young-age breast cancer tissues.

Young Average (log2)	Old Average (log2)	Fold Change	*p*-Value	FDR *p*-Value	Gene Symbol	Description
9.99	7.91	4.25	7.30 × 10^−7^	0.0099	DPYSL2	Dihydropyrimidinase-like 2
15.26	6.45	448.69	9.25 × 10^−7^	0.0099	MUCL1	Mucin-like 1
6.59	8.84	−4.76	5.35 × 10^−6^	0.0247	SFXN2	Sideroflexin 2
12.42	10.69	3.32	5.47 × 10^−^^6^	0.0247	GSN	Gelsolin
6.61	10.44	−14.28	6.71 × 10^−6^	0.0247	RANBP3L	RAN binding protein 3-like
8.63	13.13	−22.62	6.90 × 10^−6^	0.0247	ESR1	Estrogen receptor 1
10.7	8.5	4.6	1.30 × 10^−5^	0.0332	ACVR2A	Activin A receptor type IIA
5.17	9.59	−21.37	1.35 × 10^−5^	0.0332	GLYATL1	Glycine-N-acyltransferase-like 1
9.76	6.68	8.44	1.39 × 10^−5^	0.0332	TSHZ2	Teashirt zinc finger homeobox 2
9.96	6.75	9.27	2.60 × 10^−5^	0.0497	SERPINE2	Serpin peptidase inhibitor, clade E
10.1	8.77	2.52	2.63 × 10^−5^	0.0497	WDFY1	WD repeat and FYVE domain containing 1
8.41	6.55	3.63	2.78 × 10^−5^	0.0497	PCDHGB7	Protocadherin gamma subfamily B, 7

**Table 3:** The implication of the differentially expressed genes by young age ER-positive breast cancer in tumorigenesis and cancer progression.

Gene ID	Expression Status in Young-Age Breast Cancer	Fold Change	FDR *p*-Value	Significance from Previous Literature	References
DPYSL2	Up-regulated	4.25	0.0099	*DYYSL2* is a regulator of cytoskeletal dynamics in growing axons. *DPYSL2* knockout in mesenchymal-like cells inhibits cell migration, invasion, stemness features, tumour growth rate, and metastasis. Interaction between *DPYSL2* and Janus kinase 1 induces the expression of vimentin which is a marker for epithelial-mesenchymal transition (EMT) and involved in cancer progression.	[22]
MUCL1	Up-regulated	448.69	0.0099	Higher expression of MUCL1 was observed in HER2-amplified breast tumors. MUCL1 plays an essential role for MUCL1 in the proliferation of breast cancer cells, through the FAK/JNK signaling pathway.	[23]
SFXN2	Down-regulated	−4.76	0.0247	Loss of SFXN2 resulted in the accumulation of mitochondrial iron, and increased mitochondrial iron levels in TNBC generated large amounts of ROS that activated the NF-κB and TGF-β signaling pathways, which eventually promoted cell migration.	[24]
GSN	Up-regulated	3.32	0.0247	Higher expression of gelsolin was reported to be associated with axillary lymph node metastasis.	[25]
RANBP3L	Down-regulated	14.28	0.0277	In murine renal cells, loss of Ranbp3L expression resulted in the loss of epithelial characteristics, enhanced migration behavior and colony-forming capacity, and substantially altered gene expression profiles.	[26,27]
ESR1	Down-regulated	−22.62	0.0247	In estrogen receptor-positive breast cancer, a low level of ESR1 mRNA expression was a determinant of tamoxifen resistance in both adjuvant treatment and prevention settings.	[28]
ACVR2A	Up-regulated	4.65	0.0259	Over-expression of ACVR2A was associated with larger tumors (T3 and T4) in colorectal cancer clinical samples. This indicates an association between ACVR2A expression and the tumor growth process.	[29]
GLYATL1	Down-regulated	−21.37	0.0332	Down-regulated in several cancers and loss of expression was associated with higher tumor grade and poor prognosis in prostate adenocarcinoma and hepatocellular carcinoma patients.	[30,31]
TSHZ2	Up-regulated	8.44	0.0332	Up-regulation of TSHZ2 was found to repress tumor growth and metastasis and induce mammary gland development in mice.	[32]
SERPINE2	Up-regulated	9.27	0.0497	SerpinE2 overexpression in breast cancer was shown to promote metastatic spread by modulating the extracellular matrix.	[33]
WDFY1	Up-regulated	2.52	0.0497	WDFY1 positively regulated Toll-like receptor (TLR) 3 and 4 signalings. TLR signaling regulates breast cancer cell proliferation in TP53 mutated cells.	[34]
PCDHGB7	Up-regulated	3.63	0.0497	Overexpression of PCDH7 stimulated breast cancer cell proliferation and invasion in vitro and the formation of bone metastasis in vivo. PCDH7 was found to play role in bone metastasis in breast cancer.	[35]

**Table 4:** Clinicopathological characteristics of 56 breast carcinoma cases stained with GLYATL-1.

Demographic and Clinicopathological Characteristics	Frequency (Percentage)
**Age**	
Old (≥55 years)	25 (44.6)
Young (≤45 years)	31 (55.4)
**Tumor size**	
<2 cm	9 (16.1)
>5 cm	8 (14.3)
2–5 cm	30 (53.6)
Unknown	9 (16.1)
**Histopathological grade (Modified Bloom Richardson Grading system)**	
Grade 1	12 (21.4)
Grade 2	25 (44.6)
Grade 3	13 (23.2)
Unknown	6 (10.7)
**Histopathological subtype**	
Invasive carcinoma, No Special Type (NST)	48 (85.7)
Other subtypes	7 (12.5)
Unknown	1 (1.8)
**Lymph node positivity**	
Negative	18 (32.1)
Positive	29 (51.8)
Unknown	9 (16.1)
**Progesterone receptor status**	
Negative	9 (16.1)
Positive	47 (83.9)
**HER-2 expression**	
Negative	35 (62.5)
Positive	14 (25.0)
Unknown	7 (12.5)
**GLYATL-1 expression**	
High	23 (41.1)
Low	33 (58.9)

**Table 5:** Clinicopathological characteristics of 74 breast carcinoma cases stained with RANBP3L.

Demographic and Clinicopathological Characteristics	Frequency (Percentage)
**Age**	
Old (≥55 years)	41 (55.4)
Young (≤45 years)	33 (44.6)
**Tumor size**	
<2 cm	11 (14.9)
>5 cm	13 (17.6)
2–5 cm	38 (51.4)
Unknown	12 (16.2)
**Histopathological grade (Modified Bloom Richardson Grading system)**	
Grade 1	24 (32.4)
Grade 2	31 (41.9)
Grade 3	14 (18.9)
Unknown	5 (6.8)
**Histopathological subtype**	
Invasive carcinoma, No Special Type (NST)	62 (83.8)
Other subtypes	12 (16.2)
**Lymph node positivity**	
Negative	27 (36.5)
Positive	31 (41.9)
Unknown	16 (21.6)
**Progesterone receptor status**	
Negative	12 (16.2)
Positive	60 (81.1)
Unknown	2 (2.7)
**HER-2 expression**	
Negative	49 (66.2)
Positive	16 (21.6)
Unknown	9 (12.2)
**RANBP3L expression**	
High	47 (63.5)
Low	27 (36.5)

**Table 6:** Association between GLYATL-1 expression and the clinicopathological features of breast cancer patients.

Parameters	N	GLYATL-1 Expression		*p*-Value
		High	Low	
**Total, *n* (%)**	56	23 (41.1)	33 (58.9)	
**Age (years)**				0.551
≥55	25 (44.6)	10 (43.5)	15 (45.5)	
≤ 45	31 (55.4)	13 (56.5)	18 (54.5)	
**Tumor size**				0.507
<2 cm	9 (16.1)	2 (8.7)	7 (21.2)	
>5 cm	8 (14.3)	4 (17.4)	4 (12.1)	
2–5 cm	30 (53.6)	12 (52.2)	18 (54.5)	
Unknown	9 (16.1)	5 (21.7)	4 (12.1)	
**Histopathological grade**				0.574
Grade 1	12 (21.4)	5 (21.7)	7 (21.2)	
Grade 2	25 (44.6)	10 (43.5)	15 (45.5)	
Grade 3	13 (23.2)	4 (17.4)	9 (27.3)	
Unknown	6 (10.7)	4 (17.4)	2 (6.1)	
**Histopathological Subtype**				0.11
Invasive carcinoma, No Special Type (NST)	48 (15.2)	18 (78.3)	30 (90.9)	
Other subtypes	7 (67.5)	5 (21.7)	2 (6.1)	
Unknown	1 (1.8)	0 (0.0)	1 (3.0)	
**Lymph node status**				0.005
Negative	18 (32.1)	12 (52.2)	6 (18.2)	
Positive	29 (51.8)	6 (26.1)	23 (69.7)	
Unknown	9 (16.1)	5 (21.7)	4 (12.1)	
**PR status**				0.449
Negative	9 (16.1)	3 (13.0)	6 (18.2)	
Positive	47 (83.9)	20 (87.0)	27 (81.8)	
**HER-2**				0.786
Negative	35 (62.5)	13 (56.5)	22 (66.7)	
Positive	14 (25.0)	7 (30.4)	7 (21.2)	
Unknown	7 (12.5)	3 (13.0)	4 (12.1)	

**Table 7:** Association of RANBP3L expression with the clinicopathological features of breast cancer patients.

Parameters	N	RANBP3L Expression		*p*-Value
		High	Low	
Total, *n* (%)	74	47 (63.5)	27 (36.5)	
**Age (years)**				0.108
≥55	41 (55.4)	23 (48.9)	18 (66.7)	
≤45	33 (44.6)	24 (51.1)	9 (33.3)	
**Tumor size**				0.088
<2 cm	11 (14.9)	5 (10.6)	6 (22.2)	
>5 cm	13 (17.6)	7 (14.9)	6 (22.2)	
2–5 cm	38 (51.4)	24 (51.1)	14 (51.9)	
Unknown	12 (16.2)	11 (23.4)	1 (3.7)	
**Histopathological grade**				0.038
Grade I	24 (32.4)	20 (42.6)	4(18.4)	
Grade II	31 (41.9)	19 (40.4)	12 (44.4)	
Grade III	14 (18.9)	6 (12.8)	8 (29.6)	
Unknown	5 (6.8)	2 (4.3)	3 (11.1)	
**Histopathological Subtype**				0.54
Invasive carcinoma, No Special Type (NST)	62 (83.8)	39 (83.0)	23 (85.2)	
Other subtypes	12 (16.2)	8 (17.0)	4 (14.8)	
**Lymph node status**				0.202
Negative	27 (36.5)	19 (40.4)	8 (29.6)	
Positive	31 (41.9)	16 (34.0)	15 (55.6)	
Unknown	16 (21.6)	12 (25.5)	4 (14.8)	
**PR status**				0.108
Negative	12 (16.2)	9 (19.1)	3 (11.1)	
Positive	60 (81.1)	38 (80.9)	22 (81.5)	
Unknown	2 (2.7)	0 (0.0)	2 (7.4)	
**HER-2**				0.584
Negative	49 (66.2)	29 (61.7)	20 (74.1)	
Positive	16 (21.6)	11 (23.4)	5 (18.5)	
Unknown	9 (12.2)	7 (14.9)	2 (7.4)	

## Data Availability

All related data are available within the article.

## Supplementary Materials

The following supporting information can be downloaded at: https://www.mdpi.com/article/10.3390/biomedicines11010200/s1, Table S1: Statistical significance of the TCGA data comparison.
